# Visible rodent brain-wide networks at single-neuron resolution

**DOI:** 10.3389/fnana.2015.00070

**Published:** 2015-05-28

**Authors:** Jing Yuan, Hui Gong, Anan Li, Xiangning Li, Shangbin Chen, Shaoqun Zeng, Qingming Luo

**Affiliations:** ^1^Britton Chance Center for Biomedical Photonics, Wuhan National Laboratory for Optoelectronics-Huazhong University of Science and TechnologyWuhan, China; ^2^Key Laboratory of Biomedical Photonics of Ministry of Education, College of Life Science and Technology, Huazhong University of Science and TechnologyWuhan, China

**Keywords:** neural circuit, cell type, vasculature, labeling, brain-wide optical imaging, single axon

## Abstract

There are some unsolvable fundamental questions, such as cell type classification, neural circuit tracing and neurovascular coupling, though great progresses are being made in neuroscience. Because of the structural features of neurons and neural circuits, the solution of these questions needs us to break through the current technology of neuroanatomy for acquiring the exactly fine morphology of neuron and vessels and tracing long-distant circuit at axonal resolution in the whole brain of mammals. Combined with fast-developing labeling techniques, efficient whole-brain optical imaging technology emerging at the right moment presents a huge potential in the structure and function research of specific-function neuron and neural circuit. In this review, we summarize brain-wide optical tomography techniques, review the progress on visible brain neuronal/vascular networks benefit from these novel techniques, and prospect the future technical development.

## Introduction

The brain is the most sophisticated, complex and significant organ in the bodies of humans and other higher organisms. The brain is the seat of action and cognition; however, its function is hindered by various neurological and mental diseases. Thus far, the structure and functions of the mammalian brain remain largely unknown. Elucidating the structure and functions of the brain is one of the most challenging research subjects since the Human Genome Project (Petreanu et al., 2009). Characterizing the structure of the brain at high resolution is crucial for understanding its functions and dysfunction (Koch and Reid, 2012).

The brain consists of multiple fine components. The human brain generally contains approximately one hundred billion neurons and a greater number of glial cells, as well as blood vessels that travel through the densely packed cells to form extremely complex neurovascular networks. These blood vessels provide neurons and glia with energy and nutrition and clear away waste. For example, once their blood supply is blocked, neurons cease to produce action potentials within seconds and die of irreversible damage within minutes. The sizes of these brain components vary greatly: the diameter of a typical neuron is approximately 10–30 μm, and the diameters of small cells and capillaries range from 2 to 5 μm (Petersen et al., 2003; Tsai et al., 2009), whereas the diameter of a common neurite projected from soma reaches the submicron level, approximately 1 μm or less (Sun et al., 2014). Therefore, to reconstruct neural and neurovascular networks at single-neuron resolution, the imaging voxel size of the detection system should not exceed one cubic micron, which guarantees the true representation of the brain-wide neuronal and vascular systems.

The brain’s functions, including the generation of thoughts, emotions, perceptions, actions and memories, rely primarily on interconnected groups of neurons, called neuronal networks rather than on individual neurons. The brain has two types of neural circuits: local circuits, which neurites inhabit in the same brain area, and distant circuits, which neurites projecting cross long-range distance within different brain areas. Distant neural circuits possibly project from the cerebral cortex to neurons as far away as the spinal cord, whose input and output may travel throughout the whole brain (Jbabdi and Behrens, 2013). Similar to the integrated circuits of a microchip, local neural circuits are connected by the wire-like projections of long-distance neural circuits. Cooperation between local and distant circuits is vital to advanced neural functions (Miyamichi et al., 2011). Due to the interconnected, long-range nature of many neuronal networks, visualization of the brain’s structure and function must be conducted over a large volume, preferably brain-wide (Osten and Margrie, 2013).

Because of the differences between individual brains, the ideal of visualizing neural pathways and the neurovascular networks is to simultaneously detail the fine morphology of cells and blood vessels in regions of interest in high resolution. However, in higher mammals, complicated neural circuits are composed of tens of billions of neurons, rendering traditional imaging methods inadequate (Bassett and Gazzaniga, 2011; Amunts et al., 2013). Even the fine cellular-vascular architecture of the comparatively less complicated mouse brain and the interconnections within its regions remain unclear (Silvestri et al., 2013).

Using multiple labeling and imaging techniques, several brain research projects have sought to reconstruct at high resolution the fine cellular-vascular morphology of regions of interest in mammals, particularly rodents, and to provide a visualization of neural pathways and the neurovascular networks. Optical microscopy techniques, combined with advanced labeling methods, provide the best access for exploring the brain-wide neuronal/vascular networks at single-neuron resolution. Here, we review the recent advances with a focus on two aspects: (1) techniques for the brain-wide optical tomography and the matchable labeling for neuronal/vascular networks; and (2) application of brain-wide labeling and optical imaging, which include neuronal morphology, long-distance projection of the specific labeled neurons, and cytoarchitecture and vascular networks. Challenges are also discussed in the final part of prospectives.

## Brain-Wide Labeling and Optical Imaging

### Labeling the Brain for Optical Imaging

Owing to a lack of optical contrast, nervous tissue must be labeled before imaging by optical microscopy. Golgi silver staining, which was invented by Golgi and improved by Ramon y Cajal one hundred years ago, has been recognized as one of the most elegant and effective methods for distinguishing the morphology of neurons (Sotelo, 2003). Since then, many methods have been developed for staining the cytoarchitecture of the brain such as Nissl staining, in which neuronal somas are visualized by staining the rough endoplasmic reticulum (or “Nissl bodies”) (Windhorst and Johansson, 1999). To study the structure and organization of neural circuits and neurovascular networks, neuronal tracers were developed to ascertain the cell population and to analyze the neuronal anatomical connections (Garey, 1999). Standard neuronal tracers can label the entire structure of the neuron and circuit but fail to identify the interconnected cell types (Miyamichi et al., 2011). Histochemical techniques such as immunolabeling can label specific cell types but are unable to label deep structures in the intact brain (Shi et al., 2011). Therefore, histochemistry is typically performed on cultured neurons, tissue slices or superficial layers of *in vivo* tissue. To study neural circuits spanning vast volumes, these conventional methods have to be improved to label the whole brain or tissue uniformly. Luo’s group modified the Golgi-Cox (Zhang et al., 2011) and Nissl (Wu et al., 2014) staining methods and first achieved uniform whole-brain staining of rodents. Recently, whole brain immunohistochemistry has been developed to immunolabel deep tissue for volume imaging and applied to embryonic and adult brains (Chung et al., 2013; Renier et al., 2014). Golgi-Cox or Nissl staining are usually simpler and cheaper, but nonspecific for different neuronal types. Since Golgi staining is known that axons of the randomly stained neurons (less than 5%) are not always completely labeled (Binzegger et al., 2010), it is better suitable to study dendritic arbor morphologies than axonal projections. On the other hand, immunolabeling and transgenetic labeling allow specifically targeting selected neuronal populations, although with a payback of higher costs and complexity.

Transgenic and fluorescent probe labeling techniques make it possible to visualize neurons of a specific subtype and to trace neurite projections and transsynaptic circuits (Markram et al., 2004; Luo et al., 2008; Madisen et al., 2010; Huang and Zeng, 2013). To label diverse neurons in different regions simultaneously, the use of multiple fluorescent probes has been introduced to neural labeling and imaging (Shaner et al., 2005; Livet et al., 2007; Dumas et al., 2015). Neuroanatomy has been revolutionized by genetic dissection (Luo et al., 2008), which enables the systematic mapping and classification of both long-distance and local connections. However, due to the complexity of whole-brain imaging procedures, fluorescent signals are likely to be quenched, thereby compromising the detection of weak connections (Chung et al., 2013; Gong et al., 2013; Renier et al., 2014; Susaki et al., 2014; Yang et al., 2014). Efforts are being made to find a way of preserving this fluorescence based on the fact that GFP and YFP fluorescence can be recovered in an alkaline environment (Heim et al., 1995; Robey et al., 1998). Xiong et al. found that the loss of GFP or YFP fluorescence during resin embedding results from the protonation of the chromophore (Xiong et al., 2014). This result provides theoretical guidance for conserving and recovering weak fluorescence used to reveal neural circuits.

### Brain-Wide Optical Tomography

The imaging depth of traditional optical microscopy is restricted by the absorption and scattering of light in tissue. Normally, an imaging depth of only a few hundred microns can be reached by optical microscopes. The application of traditional optical microscopy is thus limited to the imaging of brain slices or of the superficial layer of the cortex. To achieve both high voxel resolution and a large detection range in three dimensions, optical microscopy and histology have to be combined to acquire serial images, from which 3D reconstructions of the brain structure can be generated. To obtain an optical tomograph of a whole rodent brain that spans several centimeters, many parameters have to be considered and balanced, such as isotropic resolution, imaging range, imaging time, robustness of the imaging system, auto-registration of the images, and expenses, et al. Several approaches have been developed, such as light-sheet illumination microscopy (LSM), serial two-photon tomography (STP) and micro-optical sectioning tomography (MOST).

#### Optical Tomography by Light-Sheet Illumination with Chemical Clearing

Dodt et al. combined LSIM (Siedentopf and Zsigmondy, 1902) and chemical clearing (Spalteholz, 1914), two 100-year-old techniques, to achieve fast imaging of transparent intact mouse brain (Dodt et al., 2007). They named it Ultramicroscopy. Side illumination is employed in parallel, and only a thin layer of brain tissue perpendicular to the optical imaging light path is illuminated. Thus, the interference from out-of-focus background light is eliminated. With this illumination scheme, wide-field imaging can be used to acquire section images at a high rate. Three-dimensional imaging of the whole brain sample can be achieved by moving the sample or by translating the illumination beam.

In recent years, many optical clearing techniques have been invented to generate transparent brain tissue so that the illumination light can pass through the entire sample. Methods including Scale (Hama et al., 2011), CUBIC (Susaki et al., 2014), SeeDB (Ke et al., 2013), 3DISCO (Ertürk et al., 2012a,b, 2014), iDISCO (Renier et al., 2014), and ClearT (Kuwajima et al., 2013) make the brain transparent by immersion in a clearing reagent. The scatters in fixed tissues are exchanged with optical clearing reagents, and then the refractive indexes of tissues become uniform to reduce the amount of light scattering. While techniques such as CLARITY (Chung and Deisseroth, 2013; Chung et al., 2013; Tomer et al., 2014) and other similar solutions (Yang et al., 2014) employ electrophoresis to remove lipids from the tissue, making the tissue transparent.

Imaging intact transparent mouse brain enables to avoid mechanical process during imaging (Kim et al., 2013). The imaging speed of wide-field LSM is much faster than those of point-scanning brain-wide optical imaging techniques. With LSM, the brain can be imaged multiple times. However, even with chemical clearing, it is still a big challenge to obtain a brain-wide tomograph with a consistent voxel resolution since the brain cannot be completely transparent, which may lead to a dramatic drop of imaging contrast and resolution somewhere deep in the brain. Structural illumination (Kalchmair et al., 2010), confocal slit detection (Silvestri et al., 2012) and a virtual-slit effect of sCMOS camera (Tomer et al., 2014) had been introduced to significantly increase image contrast also deep inside the sample. But these attempts fail to improve the axial resolution of LSM, which still limited to 10 μm. Therefore, LSM hasn’t been suitable for distinguishing and tracing axons currently and more efforts should be focused on the improvement of axial resolution.

#### Optical Tomography by Optical Sectioning with Sequential Tissue Removal

Another way to achieve deep brain optical imaging is to remove imaged brain tissue before imaging at each step. Only the superficial layer of tissue needs to be imaged, thus avoiding the limitation posed by light penetration depth. An optical sectioning scheme is used to restrain the interference from an out-of-focus background.

Tsai et al. proposed an all-optical histology (AOH) method, combining laser tissue ablation by amplified ultrashort laser pulses with two-photon excitation imaging by unamplified pulses (Tsai et al., 2003). Iterative ablation and imaging of samples lasted until the data acquisition ended. However, this method has not been demonstrated to visualize whole brain.

Ragan et al. developed the STP method, which utilizes a microtome and a two-photon excitation microscope based on mechnical scannings (Ragan et al., 2007, 2012). In the latest version, mouse brain samples were embedded in agarose and fixed to a motorized three-dimensional translation stage. At each step, two-photon imaging of the superficial layers of the tissue was performed in mosaic splicing mode. After imaging, the sample was translated to the vibratome, and the imaged layers of tissue were removed. This process was serially repeating. Simple agarose-embedded sample preparation and short data acquisition due to sparse axial sampling make STP become a useful tool for mesoscale anatomy. STP has demonstrated to obtain the specific cell-type distribution in the olfactory bulbs of transgenic mouse by imaging *z*-stack of 800 optical sections (2.5 μm *z* spacing) (Ragan et al., 2012). The image datasets covering the entire mouse brains (over 1000 samples) were acquired by STP by imaging single optical sections at a coarse axial sampling manner (50 or 100 μm *z* spacing) (Ragan et al., 2012; Oh et al., 2014).

Zheng et al. adopted another imaging strategy to increase the imaging speed of brain-wide optical tomography (Zheng et al., 2013). Acoustic-optic deflector (AOD) and sample stage had been used for fast line scanning and continuous sample motion of high-speed automated two-photon excitation microscope, respectively, which resulted in about 2.5-fold faster than the conventional mosaic imaging one (Ragan et al., 2012) using similar optical parameters. AOD enables to improve the prolonged system stability due to it is a kind of inertia-free and non-mechanical scanning device.

All of these three mentioned methods employed two-photon excitation microscopy to acquire optical sections and determine axial resolution. Two-photon excitation imaging can penetrate relatively deep into the tissue; thus, some influence of surface roughness caused by slicing on image quality can be avoided by imaging at a certain depth beneath the surface. Current point-scan serial imaging mode of two-photon excitation determines the imaging speed. Continuous running of several days for each full-volumetric whole-brain dataset acquisition asks a high demand of the prolonged system stability. Brain-wide optical tomography based on optical section has a great potential to be improved.

#### Optical Tomography by Imaging with Simultaneously Ultrathin Physical Sectioning

An alternative methodology of axial resolution improvement is to employ physical rather than optical sectioning approaches. Array tomography has been demonstrated to obtain ordered ultrathin resin-embedded sections and corresponding images. However, this approach needs manual image registration. The optimal idea is to section and image simultaneously and automatically to avoid extra registration.

McCormick et al. invented a knife-edge scanning microscopy (KESM), which features a knife-collimator assembly of providing illumination as well as the means to cut individual sections of tissue (McCormick et al., 2004; Mayerich et al., 2008). Although KESM has been tried to perform large-scale imaging of the mouse brain with a stair-step cutting, several obstacles have to be overcome before obtaining high-quality and high-resolution brain-wide image data, such as the non-uniform deformation of the fine structure, unpredictable chatter during the physical sectioning, the matched sample preparation, and so on.

Luo’s group developed a novel combination of the microscopic optical imaging and sectioning to obtain tomography (MOST) of a whole mouse brain with micrometer resolution (Li et al., 2010). MOST decouples the illumination and cutting and makes the physical sectioning more easily free of chatter. The tomographic sectioning mode in MOST is carried out column-by-column in the same layer to provide a short and constant immersion time for each layer, which avoids non-uniform deformation of the brain structure. Both KESM and MOST use the ultrathin physical sectioning to achieve uniform and high axial resolution. The specific design of MOST ultimately simplifies the optical imaging system and makes it possible to establish a prolonged stable and robust equipment since each part can be easily optimized. Combining with various traditional histological staining methods, such as Golgi (Li et al., 2010) and Nissl staining (Wu et al., 2014), MOST enabled to explore the detailed 3D map of different components in the brain at 1-micron voxel resolution. Coupled with a confocal laser scanning fluorescence imaging, a fluorescence MOST (fMOST) system has been reported. Combining a novel resin-embedding method for maintaining fluorescence, the fMOST has shown the ability to image a fluorescent protein transgenic whole mouse brain at a one-micron voxel resolution, and the long-distance pathways were traced minutely and without interruption for the first time (Gong et al., 2013). The MOST and its series have shown the robustness of consistent high-resolution imaging over a large range (Parekh and Ascoli, 2013), and provided a means to traversal each voxel in the entire brain, rather than sample limited numbers of sections.

These techniques enable brain-wide imaging at unprecedented resolution and have created opportunities for exploring and understanding the structure and functions of neuronal networks across the whole brain; these opportunities are of great interest to neuroscientists. The requirements of neuroscientists for new technologies have expedited the development of whole-brain optical imaging methods quickly from the laboratory to the user. Products employing the above-mentioned techniques have been commercialized [e.g., LSM (by Zeiss or Lavision Biotech), STP (by TissueVision) and MOST (by OeBio)]. Developments in whole-brain optical imaging will open a new chapter in neuroscience research.

## Progress in Characterizing Visible Brain-Wide Networks

### Cell Type

The brain’s complexity is exemplified by its variety of neuronal cell types (Huang, 2014; Mitra, 2014). Different types of neurons can be considered analogous to the various components of integrated electrical circuits. To understand the path of information flow in a neural circuit, the first questions we must address are as follows: How many “components” are in the brain? Despite its importance, a reasonable classification scheme for neuronal subtypes has not emerged, even after more than 100 years of debate. What is the distribution of axonal arborizations in different brain sub-regions? How many ascending and descending axons do individual neurons have? What are the relative locations of the axonal and dendritic arbors? These remain basic questions of neuroscience. Therefore, a fundamental goal of neuroscience is to acquire fine structural and positional descriptions of different neurons and to define their cell type and distribution.

#### Morphology

The diversity of neuronal subtypes is first reflected in their morphology. Whether in different brain areas or in the same brain area, the vast variation in neuronal morphologies provides an abundance of natural distinctions that facilitate neuron classification. Criteria for the classification of neuron subtypes remain in dispute (DeFelipe et al., 2013); however, morphology is widely considered one of primary criteria of cell type (Svoboda, 2011). The intact morphology of a neuron enables a dissection of its information input and output, and its spatial range and location can suggest its role in neural circuits, which determines specific function of a neuron in different brain functions (Lichtman and Denk, 2011; Parekh and Ascoli, 2013).

The imaging of neuronal morphology still currently uses traditional sectioning methods. Digital methods for the reconstruction of neuronal morphology have shifted scientists away from manually drawing to either interactively or automatically tracing (Parekh and Ascoli, 2013). Sakmann’s group systematically described excitatory neurons in the barrel cortex and interneurons in L2/3 in adult rats using the traditional method (Helmstaedter et al., 2009; Oberlaender et al., 2011, 2012). In addition, Markram’s group studied the diversity of somatosensory cortical interneurons and neurons in L1 based on neuronal morphology (Markram et al., 2004; Parekh and Ascoli, 2013; Muralidhar et al., 2014). NeuroMorpho.Org has collected published reconstructions of neuronal morphology contributed by more than 100 labs and has set up the largest open-access neuronal three-dimensional reconstruction database thus far. However, the manual acquisition approach only suits a small number of studies involving important neuronal subtypes. Acquiring the morphology of all cell types in the brain by a manual approach is an impossible task.

**Figure 1:**
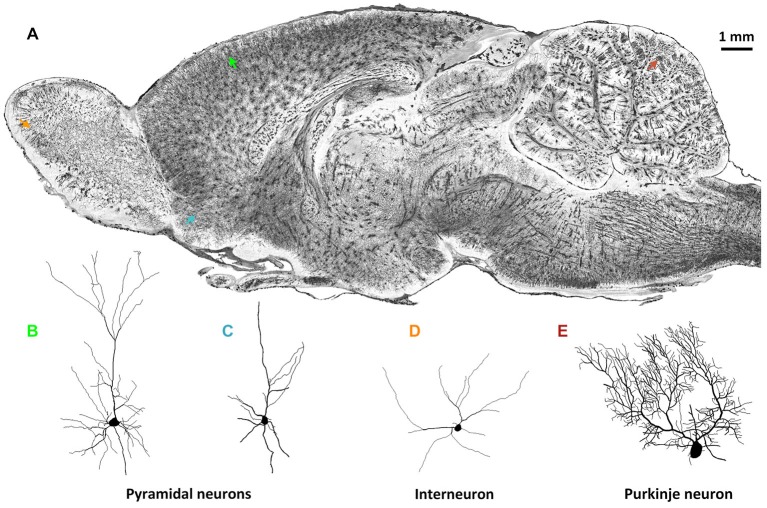
**Whole-brain imaging of rat neuronal morphology with Golgi staining (Vbn, 2015). (A)** A sagittal image of a 200 μm thickness projection from a whole-brain imaging dataset acquired by MOST method (Li et al., 2010). Arrowheads in different colors in **(A)** indicate individual neurons. **(B)** Pyramidal neuron in somato motor cortex (green in **(A)**); **(C)** Pyramidal neuron in anterior olfactory nucleus of olfactory bulb (blue); **(D)** Interneuron in glomerular layer of olfactory bulb (orange); **(E)** Purkinje cell in cerebellum (brown).

Brain-wide optical tomography capable of acquiring data on the fine morphological characteristics of neurons in the intact rodent brain would be helpful for the expansion of the current neuron morphology database. Because brain-wide optical tomography avoids the irreversible physical damage to neuronal morphology caused by the limited thickness of traditional histology sections, high-resolution brain-wide optical imaging would enable the acquisition of the 3D morphology of single intact neurons. MOST (Li et al., 2010) allowed the reconstruction of detailed cell morphology in the whole brain not only of mouse but also of rat using Golgi staining. Figure 1 shows the detailed morphology of multiple types of neurons in different brain regions in the same rat brain by the MOST method (Li et al., 2010). 3D imaging and reconstruction of neurons of various shapes in the whole brain would be helpful for codifying new morphology standards, which would permit a more systematic classification of neurons.

#### Cell Type-Specific Distributions

**Figure 2:**
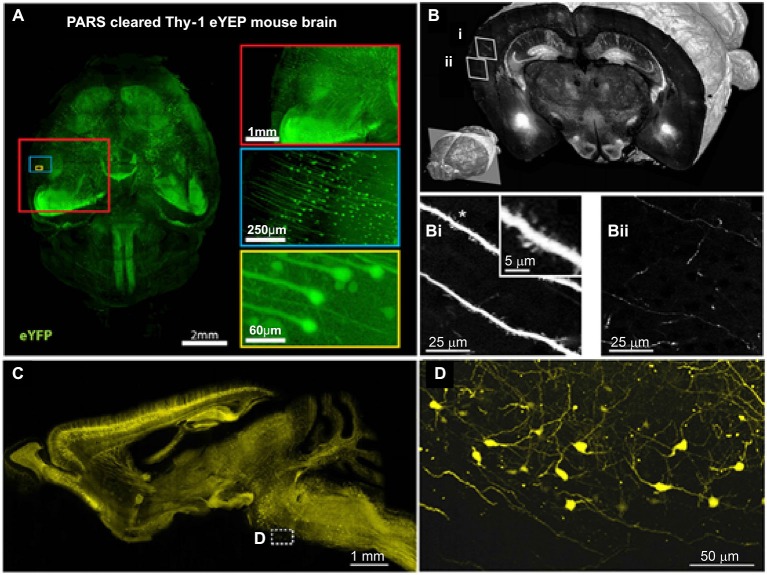
**Brain-wide distribution of Thy-1 neurons imaged by LSM, STP and fMOST. (A)** Whole-brain image (*z* = 6 mm) of adult Thy1-eYFP mouse after PARS clearing for 10 days. The boxes on the right show high-magnification images of indicated areas. PARS, perfusion-assisted agent release *in situ*. Taken from Yang et al. (2014). **(B)** 3D view of a coronal section of the GFP brain. Lower left: position of the coronal plane in the imaged mouse brain (approximately 2.5 mm from Bregma). Enlarged views demonstrating visualization of dendritic spines **(Bi)** and fine axon fibers **(Bii)**. Adapted from Ragan et al. (2012). **(C)** Sagittal image reconstructed from a stack of virtual sagittal sections (thickness: 50 μm) of an adult Thy1-eYFP mouse brain. **(D)** Higher magnification views of some soma and fine neurites in the white box of **(C). (C)** and **(D)** redrawn from the imaging dataset published in Gong et al. (2013).

To analyze the organization and signal processing mechanisms of the nervous system, knowledge of the spatial distributions of specific cell types is fundamental. The distributions of specific neuronal population, such as acetyl cholinergic (Armstrong et al., 1983), dopaminergic (Björklund and Dunnett, 2007) and serotonergic neurons (Fu et al., 2010; Russo and Nestler, 2013), have been analyzed using traditional methods; however, these studies were limited primarily to local areas. Recently, the projection, location and distribution of specific subtypes of interneurons (Weissbourd et al., 2014) and of serotonergic neurons (Pollak Dorocic et al., 2014) have been characterized in the mouse brain with manually sectioning and imaging. To facilitate mapping of whole brain distribution patterns, automatic imaging techniques have been combined with cell type-specific markers. Using these approaches, we can investigate the distribution and specific connections of neurons at the bulk volume level. For example, STP (Ragan et al., 2012), fMOST (Gong et al., 2013) and LSIM techniques (Chung et al., 2013) have revealed the distribution of Thy-1 neurons (expressed GFP or YFP) and the projection patterns of their neuritis (Figure 2). In addition, the whole-brain distribution of somatostatin-positive interneurons (Taniguchi et al., 2011) was revealed. These methods have provided an unprecedented level of information on the spatial distribution of specific neuronal types and of circuits, which is necessary to further understand the functions of the central nervous system.

### Mesoscale Brain-Wide Neuroanatomical Connectivity

Complex brain functions are constrained and defined by neural circuits formed by numerous interconnected neural cells. Thus far, knowledge of intricate neuroanatomical circuitry has remained extremely sparse. To this end, a project dubbed the “mesoscale brainwide neuroanatomical connectivity of the mouse” has been undertaken by the neuroscience community (Bohland et al., 2009).

#### Brain Region-Level Connectivity

In recent decades, both fMRI and diffusion tensor imaging have promoted our understanding of the structure and functions of the nervous system at the macroscale. The BOLD signal defined functional connectivity and diffusion tensor determined that white matter tracts represent an indirect description or gross representation of neural circuits, missing finer-scale information such as the locations of axons, dendrites and synapses (Silasi and Murphy, 2014). In the 21st century, a combination of advanced anatomical tract tracing and optical microscopy has provided powerful tools for dissecting neural circuits (Osten and Margrie, 2013). Initially, the study of neural circuits in rodents primarily focused on local circuits or on specific functional systems in small regions (Petersen et al., 2003). Zingg et al. applied anterograde and retrograde tracers and section imaging to a systemic study of intracortical connections. They revealed that the whole mouse cortex consists of 8 subnetworks with unique topologies (Zingg et al., 2014). However, the manual sectioning and reconstruction of the brain is time consuming and labor intensive, making it unsuitable for the study of brain-wide neural circuits.

The invention of automatic whole brain imaging has allowed large-scale and systematic studies of detailed neuronal connections between different brain regions. For example, Ragan et al. exploited the STP technique to acquire high-throughput fluorescence imaging (Ragan et al., 2012). This method supplies an efficient tool for routine neuroanatomy studies in mouse models. Oh et al. made advantage of automation and the high-throughput nature of STP to trace axonal projections throughout the brain from defined regions, generated a whole-brain connectivity matrix and demonstrated the network properties to be small-world and scale-free (Oh et al., 2014). All the 469 injected brains and 295 non-overlapping target regions were investigated to make quantitative analysis on connections. This study presents an unprecedented region-level connectivity in different regions across whole brain (Oh et al., 2014).

#### Neurite-Level Connectivity

Neuroscientists have been attempting to map detailed neural circuits to better understand the relationship between the structure and functions of the brain (Lichtman and Denk, 2011). For example, researchers have found that many olfactory sensory neurons in the mouse are characterized by the expression of one of approximately 1,400 odorant receptors defined by genes (Ghosh et al., 2011). A genetically defined class of olfactory sensory neurons has convergent axonal projections to two glomeruli at mirror symmetric locations in the olfactory bulb, forming an odor map (Miyamichi et al., 2011). However, axons from individual glomeruli project diffusely to the olfactory cortex without an apparent spatial order (Sosulski et al., 2011). The long-range connections from the olfactory bulb to the olfactory cortex remain unclear and are an impediment in the study of olfaction (Miyamichi et al., 2011).

Similar to integrated electrical circuits, which are composed of different components, neural circuits consist of extensively interconnected neuronal cells. To study the information flow within neural circuits, the complete structure of neurons, together with axons and dendrites, in the whole brain must be resolved at high resolution. This study requires single-neuron resolution imaging of the whole brain. The STP method is capable of good resolution in the transverse x-y plane but has a large interval between axial sections, leading to incomplete 3D datasets, which cannot be used to decipher real pathways and connections within neural circuits. Even with the advancement of optical clearing, LSM cannot image clear and complete long-range neural circuits deep in the mouse brain.

**Figure 3:**
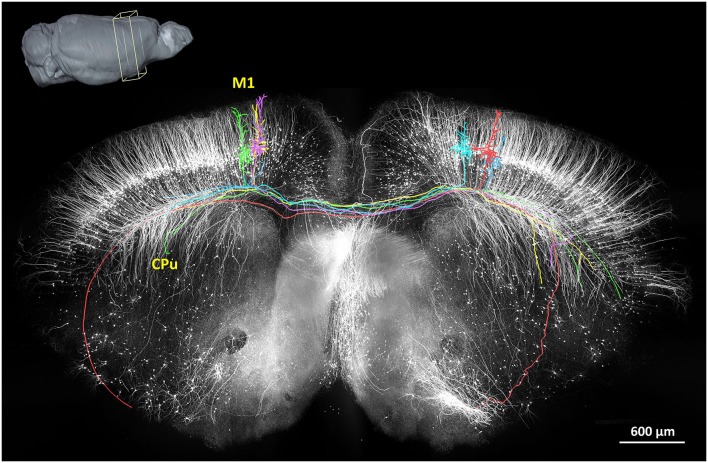
**The reconstruction of the coronal plane imaging (300 μm thick) of GFP-M line mouse brain using fMOST**. A 3D reconstruction of the mouse brain is in the top left corner; the cube at the coronal plane presents the spatial location of the data at the center. Six individual pyramidal neurons were segmented and traced, indicated in different colors. Axon arbors extend in the ipsilateral hemisphere and to the caudate putamen in the contralateral hemisphere via the corpus callosum. Redrawn from the imaging dataset published in Gong et al. (2013).

In 2013, the fMOST method had demonstrated the first long-range tracing of individual axons in the mouse brain (Gong et al., 2013; Osten and Margrie, 2013). These findings not only confirmed previously discovered pathways but also confirmed several unreported, putative projection pathways. Thus far, this has been a valuable technique to provide sufficiently intricate data describing the neural circuits in an arbitrary region of the mouse brain. A representative projection pattern of homotopic axons originating from the primary motor cortex is shown in Figure 3. Hopefully, the combination of fMOST and newly developed trans-synaptic tracing methods will provide new insight into acquiring long-range and trans-synaptic tracking of specific-labeled neural circuits. Without doubt, these methods will accelerate the characterization of information flow in brain circuits.

Based on the automation of advanced optical microscopy, the current study of mesoscale brain-wide neuroanatomical connectivity of the mouse provides greater benefits than conventional manual neuroanatomy. A more complete knowledge of the local and long-range connections exhibited by neural circuits will help us to understand fundamental brain functions and neural diseases.

### Simultaneous Acquisition of Multiple Types of Information from the Brain

#### Simultaneous Visualization of the Structure and Location of Neurons

Simultaneously acquiring the structural and locational information describing neural circuits is a key issue in understanding how neural systems organize and process information. Nissl staining is the gold standard for the anatomical location of neurons in the brain. Many reference atlases based on Nissl staining have been established and widely used in anatomical studies (Dong, 2008; George Paxinos, 2012). However, due to differences between various mouse lines and different developmental stages, precisely matching every brain slice to a reference atlas is impossible. Accurately identifying structures and referencing anatomical locations remain challenging (Toga, 1999; Simmons and Swanson, 2009). Locating the projections of neurites and subnets of neural circuits in 3D space is even more difficult. Researchers have been attempting to acquire specific cell type distributions, neural connections and location information in a single intact brain using a combination of whole brain imaging and nuclear staining (Oh et al., 2014; Susaki et al., 2014), although a reference providing simultaneously collected neural circuit and anatomical data in one animal at high resolution needs to be established.

#### Acquiring Cell-Vascular Information Simultaneously

**Figure 4:**
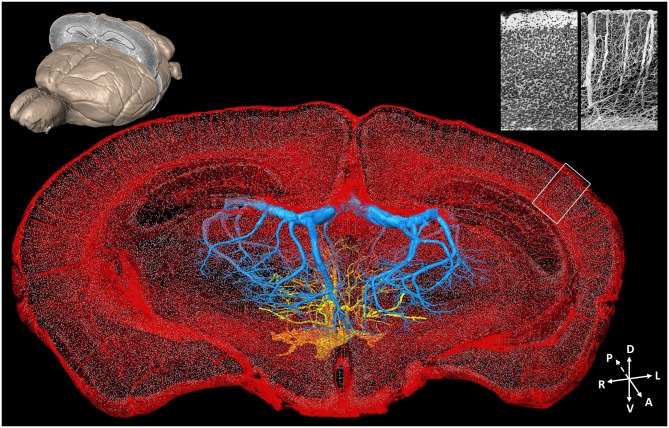
**Whole-brain cytoarchitecture and vascular networks acquired by the MOST method**. A 3D reconstruction of the mouse brain is in the top left corner; the selected 300 μm slab at the coronal plane presents the spatial location of the data at the center. The center shows the cytoarchitecture and vascular network, simultaneously acquired in the brain. Blue and yellow represents the branches of the longitudinal hippocampal vein and some thalamo-perforating arteries in thalamus, respectively, red represents all else vessels in this data set, and gray dots represent the center of somas. The enlarged views of the cytoarchitecture and vascular architecture of the white rectangle in cortical region in the data at the center are in the top right corner. Redrawn from the imaging dataset published in Wu et al. (2014).

Acquiring data on detailed neurovascular networks simultaneously with other types of neural data is essential to understanding the mechanisms of energy metabolism in the central nervous system. Based on computed tomography (CT) technology and magnetic resonance imaging (MRI), the brain-wide live imaging and localization of large vessels have been achieved at 20 μm isotropic resolution (Dorr et al., 2007, 2008). A combination of optical imaging and perfusion is common for visualizing the vessels. Kleinfeld et al. conducted vascular network imaging with 1 μm resolution in multiple small brain regions but were limited by the imaging field and depth of traditional two-photon imaging (Blinder et al., 2013). Images of large vessels in the whole mouse brain were acquired at a resolution of 3–5 μm with LSM (Hashimoto et al., 2008; Ertürk et al., 2012a). Nissl staining also provides contrast images of vessels in the neural tissue. The cytoarchitecture and vascular network data can be acquired simultaneously with unprecedented details of both individual cells and blood vessels, including capillaries (Wu et al., 2014) from the mouse brain by combining the MOST technique and a modified whole-brain Nissl staining method (Figure 4). It raises the possibility of imaging vascular networks and neural circuits as well as corresponding landmarks of brain regions/nuclei in the whole brain simultaneously.

## Perspectives

In recent years, brain-wide optical tomography has been well developed, promoting the acquisition of neural anatomical information at single-neuron resolution. However, much remains to be explored in the complicated brain. Additionally, rodent models have obvious limitations in terms of understanding the advanced functions and dysfunctions of the human brain and the corresponding drug research. We are sure that non-human primates are better models to deal with the question, and even humans. Therefore, future studies must be conducted in a systematic and multi-disciplinary way and must include sample labeling and imaging techniques, as well as methods for processing big image data.

Optical labeling and imaging techniques that enable the fine structural characterization of neuronal networks from a large volume of brain tissue with high accuracy and efficiency still present a challenge. During the imaging process, the somas of marked neurons emit much more light than their axons and dendrites. To reduce the influence of the high intensity of light coming from the soma, the neuron must be marked sparsely, while the axons and the dendrites must contain stable, high-intensity fluorescence. Then the precious signal must remain strong until all digitized intensity distribution is recorded. Meanwhile, developing sectioning and imaging techniques with better SNR and spatial resolution is urgently needed. The resolution of whole-brain optical imaging technology should be improved from one μm to several hundred nms and may even break the optical diffraction limit. Moreover, to study the decimeter-sized primate brain, available effective specific labeling techniques, such as CLARITY (Chung et al., 2013), iDISCO (Renier et al., 2014) and CUBIC (Susaki et al., 2014), need to be optimized, and more effective novel labeling techniques need to be developed. On the other hand, imaging techniques currently used for rodents must be improved to get the faster speed, the larger detection range and the more robust performance.

With the increasing imaging resolution and sample volume, massive amounts of data are being obtained at an unprecedented rate, exceeding the processing capability of current hardware and software technologies, which have contributed to the increasing problem of “big data”. The 3D mouse brain data set in Li et al. (2010) is 8 TB (1 TB = 1024 GB), equaling the total storage capacity of ten thousands of DVD disks. Once the 3D reconstruction of whole human brain is completed in the future, the resulting data will total 8 PB (1 PB = 1024 TB). As the primary bottleneck limiting of neuroscience studies, “big data” have been widely noted for the challenge these data pose to all current methods of data storage, image processing and analysis, and data management and sharing (Akil et al., 2011). Recently, more efforts have been attempted to fully automatize image processing and information extraction (Latorre et al., 2013; Quan et al., 2013, 2014; Frasconi et al., 2014). At the end of 2014, Nature Neuroscience, with the theme “Focus on Big Data”, indicated that, neuroscientists have to learn to manage and take advantage of the big waves of data that are being generated. Fully automated methods will facilitate neuroscientists to handle the big data.

Establishing more effective research strategies is a necessity, in addition to considering the scientists, research facilities, and methods and techniques devoted to this area. Referring to the Human Genome Project, international cooperation under the guidance of the roadmap presents an important alternative to independent project teams. The Allen Institute for Brain Science in the USA has already made many beneficial attempts, starting in 2003. In addition, the integration and application of current knowledge is another area that should be a focus (Akil et al., 2011). In recent years, the 3D brain atlas database and search engines opened to the public via the internet have sprung up.

The neuroscience boom is well underway. How are neural circuits organized? How are they interconnected? How many types of neurons exist in the brain? These questions can be addressed by the interdisciplinary cooperation between researchers in the fields of neuroscience, optics, engineering, chemistry, computer science and statistics.

## Conflict of Interest Statement

The authors declare that the research was conducted in the absence of any commercial or financial relationships that could be construed as a potential conflict of interest.
